# β-hydroxybutyrate administration improves liver injury and metabolic abnormality in postnatal growth retardation piglets

**DOI:** 10.3389/fvets.2023.1294095

**Published:** 2023-11-03

**Authors:** Chengming Wang, Nan Wang, Yuankun Deng, Andong Zha, Junyao Li, Bie Tan, Ming Qi, Jing Wang, Yulong Yin

**Affiliations:** ^1^College of Animal Science and Technology, Hunan Agricultural University, Changsha, Hunan, China; ^2^Yuelushan Laboratory, Changsha, Hunan, China; ^3^Institute of Yunnan Circular Agricultural Industry, Puer, Yunnan, China

**Keywords:** β-hydroxybutyrate, liver injury, lipid, postnatal growth retardation, piglets

## Abstract

Abnormal hepatic energy metabolism limits the growth and development of piglets. We hypothesized that β-hydroxybutyrate (BHB) might improve the growth performance of piglets by maintaining hepatic caloric homeostasis. A total of 30 litters of newborn piglets were tracked, and 30 postnatal growth retardation (PGR) piglets and 40 healthy piglets were selected to treat with normal saline with or without BHB (25 mg/kg/days) at 7-d-old. At the age of 42 days, 8 piglets in each group were sacrificed, and serum and liver were collected. Compared with the healthy-control group piglets, PGR piglets showed lower body weight (BW) and liver weight (*p* < 0.05), and exhibited liver injury and higher inflammatory response. The contents of serum and hepatic BHB were lower (*p* < 0.05), and gene expression related to hepatic ketone body production were down-regulated in PGR piglets (*p* < 0.05). While BHB treatment increased BW and serum BHB levels, but decreased hepatic BHB levels in PGR piglets (*p* < 0.05). BHB alleviated the liver injury by inhibiting the apoptosis and inflammation in liver of PGR piglets (*p* < 0.05). Compared with the healthy-control group piglets, liver glycogen content and serum triglyceride level of PGR piglets were increased (*p* < 0.05), liver gluconeogenesis gene and lipogenesis gene expression were increased (*p* < 0.05), and liver NAD^+^ level was decreased (*p* < 0.05). BHB supplementation increased the ATP levels in serum and liver (*p* < 0.05), whereas decreased the serum glucose, cholesterol, triglyceride and high-density lipoprotein cholesterol levels and glucose and lipid metabolism in liver of PGR piglets (*p* < 0.05). Therefore, BHB treatment might alleviate the liver injury and inflammation, and improve hepatic energy metabolism by regulating glucose and lipid metabolism, thereby improving the growth performance of PGR piglets.

## 1. Introduction

Growth retardation (GR) is when an individual grows significantly slower than a healthy individual, accompanied by metabolic disorders, systemic inflammation, or intestinal dysbiosis, usually occurring in the early stages of life (1). Growth retardation can be divided into two categories: intrauterine growth retardation (IUGR) and postnatal growth retardation (PGR). In mammals, the incidence of IUGR is high in multiple animals such as pigs, and 15–20% of newborn piglets weigh less than 1.1 kg (2). PGR piglets are defined as piglets with normal birth weight, while the body weight during postnatal growth and development is lower than 70% of the average weight of health piglets in the same period, and there is no obvious trauma (3). The incidence of PGR piglets during lactation and after weaning is about 10–30 and 5% respectively, which seriously restricts the breeding efficiency (4). Normally, IUGR is mainly caused by decreased blood flow and insufficient supply of nutrients that are induced by uterine-placental disorders during maternal pregnancy, resulting in impaired growth and development of the fetus or its organs (5). PGR is due to the impairment of intestinal barrier function and the imbalance of intestinal microorganisms after birth that triggered a decrease in the absorption capacity of intestinal nutrients, which induced the slow growth and development of neonatal piglets (6). Interestingly, our tracing test of piglets (from birth to 60 d of age) found that some IUGR piglets were able to develop catch-up growth at weaning, whose body weight exceeded the PGR piglets (unpublished data). Our previous studies showed PGR piglets have dysregulated nutrition absorption and aberrant energy metabolism (6–9), which suggested that increasing nutrient utilization and maintaining the balance of energy metabolism might improve the growth inhibition of PGR piglets.

The liver serves as a key modulator in nutrition metabolic homeostasis involving energy storage, xenobiotic metabolism, and detoxification activity (10). Studies have shown that the metabolism of nutrients in the liver of IUGR piglets is abnormal, which inevitably impairs liver function (5, 11). We previously observed a decreased peroxisome proliferator-activated receptor gamma (PPARγ) signaling pathway and an impairment in glucose utilization in the small intestine of PGR piglets compared to these in the healthy piglets (7). These considerations raised the possibility that there is a deficient hepatic energy metabolism in PGR piglets and mediating the energy metabolism might promote their growth performance. The ketone bodies, especially β-hydroxybutyrate (BHB), are synthesized in the liver from acetyl-CoA derived primarily from fatty acid oxidation. In the case of nutritional deficiency, BHB can be used as an alternative energy source to maintain caloric homeostasis (12). Meanwhile, BHB also acts as a signaling molecule for modulating lipolysis, oxidative stress, and neuroprotection. Studies have found that mice with ketogenic insufficiency and fed a high-fat diet exhibit abnormal hepatic glucose and lipid metabolism to induce mitochondrial dysfunction and liver injury (13). BHB could prevent liver ischemia–reperfusion injury and oxidative stress by up-regulating FOXO1 and HO-1, and reduce inflammatory response and apoptotic cell death by down-regulating NF-κB and NLRP3 inflammasome (14, 15). In addition, BHB ameliorates endoplasmic reticulum stress by activating AMPK and inhibits NLRP3 (16–18).

Therefore, we hypothesized that supplementing with exogenous BHB could improve the growth performance of PGR piglets by regulating liver energy metabolism. The effect of supplementation of BHB on the liver morphology, hepatic inflammatory response and energy metabolism was determined in healthy or PGR piglets.

## 2. Materials and methods

All animals used in this study are humanely managed in accordance with Chinese animal welfare guidelines. The experimental scheme was approved by the Animal Protection and Utilization Committee of Hunan Agricultural University (Changsha, China; 2,021,042).

### 2.1. Animal and experimental design

30 litters of newborn piglets with similar parity (3rd ~ 5th parity) and genetic background (about 12 piglets per litter) were recorded. All piglets are breast-fed normally. Diet of sow and piglet, drinking water and feeding environment are carried out in accordance with the operating standards of the company’s farm. All newborn piglets are marked with ear defects. The birth weight of newborn piglets was recorded and defined as IUGR piglets according to the birth weight lower than 1.1 kg (2). At 7 days old, PGR piglets were defined according to the standard that whose body weight was less than 70% of the average weight of healthy piglets and there was no obvious trauma (8). Forty normal healthy piglets and thirty PGR piglets were randomly divided into 4 groups: control group, BHB group, PGR group and PGR + BHB group. The piglets of control and PGR group were fed daily with 5 mL saline (0.9%) from 7 days old, the piglets in BHB group and PGR + BHB group while fed with BHB solution dissolved in 5 mL saline (25 mg/kg/days, body weight was calculated according to the average weight per week). At the age of 42 days, 8 piglets in each group were randomly selected for slaughter sampling. The body weight of piglets is shown in Table 1.

**Table 1:** The body weight of healthy and post-natal growth retardation (PGR) pigs.

Item	Healthy-control	Healthy-BHB	PGR-control	PGR-BHB	*p*-value
Day 7 weight	2.83 ± 0.16^a^	2.65 ± 0.07^a^	1.83 ± 0.14^b^	2.02 ± 0.05^b^	0.000
Day 42 weight	10.44 ± 0.42^b^	13.03 ± 0.76^a^	6.35 ± 0.79^c^	9.73 ± 0.66^b^	0.000

BHB, β-hydroxybutyrate; PGR, postnatal growth retardation. ^a, b, c^Values within a row with different superscripts differ significantly (*p* < 0.05). *n* = 8 per treatment group.

### 2.2. Sample collection

After 12 h fasting, all piglets were collected the anterior vena cava blood in the blood vessels containing heparin sodium. 3,000 g was centrifuged at room temperature for 10 min and stored in the refrigerator at −80°C. After the blood was collected, the liver was taken for weighing, and then the liver sections (about 1 cm^2^) and liver samples (about 5 g) were rinsed thoroughly with cold PBS (PH 7.4) to remove blood contamination. Liver slices were quickly fixed in 4% paraformaldehyde and prepared for embedding and slicing for hematoxylin–eosin (HE) and periodate-Schaefer (PAS) staining. Liver samples were rapidly frozen in liquid nitrogen and stored at −80°C for further analysis. All samples were collected within 15 min after execution.

### 2.3. Analysis of serum biochemical indexes

Serum biochemical indexes including triglyceride (TG), cholesterol (TC), high-density lipoprotein cholesterol (HDL-C), low-density lipoprotein cholesterol (LDL-C) and glucose (GLU) were detected by automatic biochemical analyzer BS-200 (BeckmanCX4, Beckman Coulter Inc.). The specific kit was purchased from Shanghai Kehua Bio-Engineering Co., Ltd.

### 2.4. Determination of transaminase activity and BHB content in plasma and liver

The activities of aspartate aminotransferase (AST) and alanine aminotransferase (ALT) in plasma and liver were determined by commercial kit (Beijing Solarbio Science & Technology Co., Ltd.). The content of BHB in serum was detected by pig enzyme-linked immunosorbent assay (Shanghai Fanke industrial Co., Ltd.), and the content of BHB in liver was detected by β-hydroxybutyrate colorimetric assay kits (Cayman Inc., United States) according to the manufacturer’s instructions.

### 2.5. Determination of inflammatory cytokines in liver

Liver samples were homogenized in cold PBS. The liver homogenate was taken and centrifuged at 4°C for 12,000 g 10 min. The supernatant was collected for further detection. The concentrations of interleukin-1β (IL-1β), interleukin-10 (IL-10), tumor necrosis factor-α (TNF-α) and interferon-γ (IFN-γ) were detected by enzyme-linked immunosorbent assay kit (CSB-E06782p, CSB-E06779p, CSB-E16980p, CSB-E06794p, CUSABIO, https://www.cusabio.com/). The final results were normalized with the total protein concentration in each sample, and the protein concentration was detected by BCA protein concentration determination kit (Beyotime Biotech. Inc.).

### 2.6. Determination of energy metabolism index of liver

The content of adenosine triphosphate (ATP) (Jiangsu Meimian Industrial Co., Ltd., Yancheng, China) was detected by an enzyme-linked immunosorbent assay kit in pigs. The contents of oxidized coenzyme I (NAD^+^) and reduced coenzyme I (NADH) were determined according to the operating instructions of the corresponding kits (Beyotime Biotech. Inc.). The final results were normalized with the total protein concentration in each sample.

### 2.7. Quantitative reverse analyzes

According to the primer design principles, primer premier 6.0 software was used to design primers, which were synthesized by Beijing Tsingke Biotech Co., Ltd. The primer sequence is shown in Table 2. TRIZOL reagent (Invitrogen, Carlsbad, CA, United States) extracted total RNA from liver. The RNA integrity, quality and purity of the samples were determined by 1% agarose gel electrophoresis and automatic nucleic acid/protein analyzer. Using the Evo M-MLV reverse transcription Premix kit (AG11728, Accurate Biotechnology (Hunan) Co., Ltd., Chang Sha, China) reverse-transcribed RNA into cDNA. Refer to the SYBR Green Pro Taq HS Premixed qPCR Kit instructions (AG11701, Accurate Biotechnology (Hunan) Co., Ltd., Chang Sha, China), using a fluorescent quantitative PCR instrument (LightCycler®480 Real-Time PCR System, Roche, Switzerland) for real-time fluorescence quantitative PCR detection. The experiment was carried out with a reaction system of 10 μL, and each reaction was repeated three times. Refer to *Qi Ming* et al. for the reaction procedure (8). β-actin was the internal reference gene, and 2^-ΔΔCt^ was used to calculate the mRNA relative expression of the target gene.

**Table 2:** Primers used for quantitative reverse transcription-PCR.

Gene	Gene Bank No.	Sequence (5′-3′)
β-actin	XM_0031242803	F:CTGCGGCATCCACGAAACT
		R:AGGGCCGTGATCTCCTTCTG
NLRP3	NM_001256770.2	F:CCTTCAGGCTGATTCAGGAG
		R:GACTCTTGCCGCTATCCATC
IL-1β	NM_214055.1	F:CAGCCATGGCCATAGTACCT
		R:CCACGATGACAGACACCATC
IL-4	NM_214340.1	F:CCCGAGTGTCAAGTGGCTTA
		R:TGATGATGCCGAAATAGCAG
IL-8	NM_213867.1	F:GCTCTCTGTGAGGCTGCAGTTC
		R:AAGGTGTGGAATGCGTATTTATGC
IL-10	NM_214041.1	F:GGGCTATTTGTCCTGACTGC
		R:GGGCTCCCTAGTTTCTCTTCC
Bcl-2	NM_214285.1	F:AGGGCATTCAGTGACCTGAC
		R:CGATCCGACTCACCAATACC
Bax	XM_003127290.5	F:GAAGTTGAGCGAGTGTCT
		R:AGTTGAAGTTGCCGTCAG
Caspase-3	NM_214131	F:CGTGCTTCTAAGCCATGGTG
		R:GTCCCACTGTCCGTCTCAAT
Caspase-8	XM_021074712.1	F:CGAAGACCAGAGTTTGCCCT
		R:GGATCCTCACCGTGGCAG
Caspase-9	XM_013998997.2	F:GTACCCACCACCAAGGTCTG
		R:AAGCTCACGGTTCAGCAGAG
HMGCL	XM_005656051.3	F:GTCACCACGTCGTCCCAGAG
		R:TTACTGTCGCCATCTTGCCC
HMGCS2	XM_021089968.1	F:CACGACGGGGCAACTCTC
		R:AAAACCTTTGGTGGGCTGCT
BDH1	XM_021070089.1	F:TCGGGGCAGAGTCTCCTTT
		R:GCCATAAGAGGCAGAGTGGT
PCK1	NM_001123158.1	F:TAAAGCTGGGAGGTTCTGCC
		R:CCAAGGTCGCCTACGTTTTC
PGC-1α	NM_213963.2	F:GGACTGACATCGAGTGTGCT
		R:TGAGTCCACCCAGAAAGCTG
G6PC	NM_001113445.1	F:CAGGACTCCCAGGATTGGTTC
		R:ATCACAGCTACCCAGAGGAGT
C/EBP-α	XM_003127015.4	F:GGTGGACAAGAACAGCAACGA
		R:TGGTCAGCTCCAGCACCTTC
SREBP1	NM_214157.1	F:GCAAGGCCATCGACTACATC
		R:AGGTTCTCCTGCTTGAGCTT
ACC	XM_021066238.1	F:ATGAAGGCTGTGGTGATGGA
		R:CTTGGTGACTTGAGCGTGAG
SCD1	NM_213781.1	F:TAAACAGTGCTGCCCACCTA
		R:AGGGAAAGGTGTGGTGGTAG
PPARγ	NM_214379.1	F:ATTTACACCATGCTGGCCTC
		R:GGGCTCCATAAAGTCACCAA

### 2.8. Statistical analysis

All the data in this study are obtained by using Excel 2019 after preliminary finishing. All statistical analyzes were performed by one-way ANOVA using SPSS software 20.0 (SPSS Inc., Chicago, IL, United States). The differences among treatments were evaluated using Turkey’s test. All data are expressed as the mean ± standard error (SEM) of the average. *p* < 0.05 indicates that there is a significant difference between the two groups.

## 3. Results

### 3.1. Effect of BHB on liver morphology and hepatic inflammation of PGR piglets

**Figure 1:**
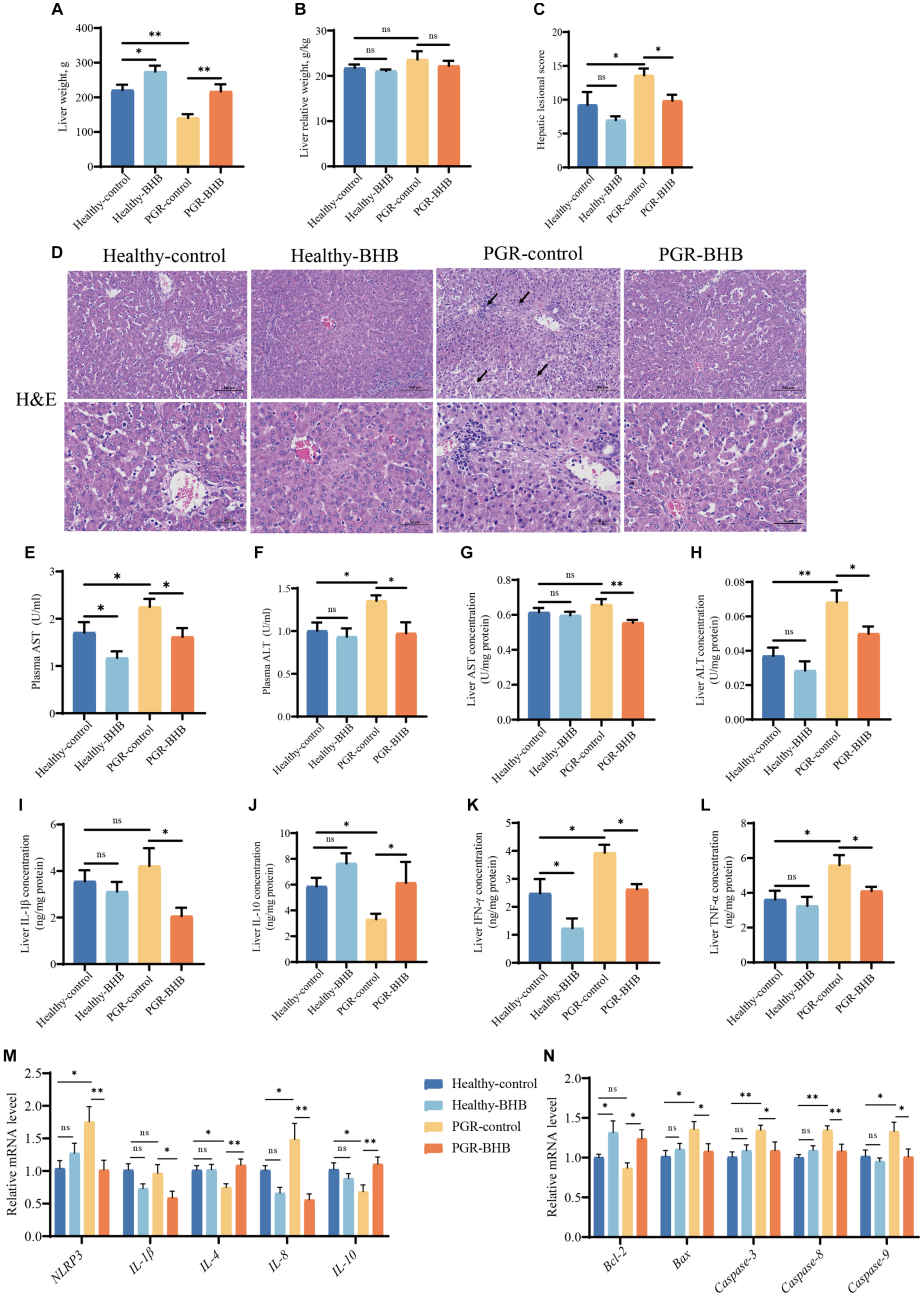
Effect of BHB on liver morphology and function of PGR piglets. **(A)** Liver weight, **(B)** liver relative weight, **(C,D)** representative images of H&E staining and lesioned score in liver, **(E,F)** plasma activity of AST and ALT, **(G,H)** liver concentration of AST and ALT, **(I–L)** liver concentration of IL-1β, IL-10, IFN-γ, and TNF-α, (**M,N**) hepatic mRNA expression of *NLRP3*, *IL-1β*, *IL-4*, *IL-8*, *IL-10*, *Bcl-2*, *Bax*, *Caspase-3*, *Caspase-8*, and *Caspase-9*. BHB, β-hydroxybutyrate; PGR, postnatal growth retardation; AST, aspartate aminotransferase; ALT, alanine aminotransferase; IL-1β, interleukin-1β; IL-10, interleukin-10; TNF-α, tumor necrosis factor-α; IFN-γ, interferon-γ; NLRP3, NOD-like receptor pyrin domain 3; Bcl-2, B-cell lymphoma 2; Bax, Bcl-2-associated X protein; Caspase-3, cysteine-aspartic acid protease 3; Caspase-8, cysteine-aspartic acid protease 8, Caspase-9, cysteine-aspartic acid protease 9. Data were presented as mean ± SEM (*n* = 8). Different lowercase letters indicated significant differences between the compared groups (*p* < 0.05).

The body weight (BW) and absolute liver weight of PGR piglets was significantly lower than those of age-matched healthy piglets (*p* < 0.05) (Table 1 and Figure 1A). Administration of BHB significantly increased the BW and absolute liver weight of healthy piglets and PGR piglets (*p* < 0.05) (Table 1 and Figure 1A), but had no significant effect on relative liver weight (*p* > 0.05) (Figure 1B).

The hepatic morphology was showed in Figure 1D, the portal vein cell necrosis, nuclear fragmentation, macrophage vacuolation, hepatocyte cord disorder, inflammatory cell infiltration, and hepatocyte watery degeneration were observed in the PGR piglets. While BHB administration in PGR piglets could alleviate their hepatic injury (*p* < 0.05) (Figures 1C,D). The activities of ALT and AST in plasma and the content of ALT in liver tissue of PGR-control piglets were significantly higher than those of healthy-control piglets (*p* < 0.05) (Figures 1E–H). As compared to the PGR-control group, BHB decreased the AST and ALT levels in the plasma and liver of PGR piglets, while BHB declined the plasma AST levels of healthy piglets in comparison with the healthy-control group (*p* < 0.05) (Figures 1E–H). Meanwhile, PGR-control group showed higher IFN-γ and TNF-α concentrations, as well as *NLRP3* and *IL-8* mRNA expression levels in the liver, but lower hepatic IL-10 concentration, and *IL-4*, *IL-10* mRNA expression levels as compared to the healthy-control group (*p* < 0.05) (Figures 1I–M). BHB treatment in healthy piglets declined the hepatic IFN-γ concentration as compared to the healthy-control group (*p* < 0.05) (Figure 1K), while BHB treatment in PGR piglets significantly decreased IL-1β, IFN-γ and TNF-α concentrations, as well as the mRNA expression levels of *NLRP3*, *IL-1β* and *IL-8* in the liver (*p* < 0.05) (Figures 1I–M). Compared to the PGR-control group, BHB treatment in PGR piglets also showed higher IL-10 concentrations, and *IL-4*, *IL-10* mRNA levels in the liver (*p* < 0.05) (Figures 1I–M). In addition, the relative expressions of *Bax*, *Caspase-3*, *Caspase-8*, and *Caspase-9* mRNA in the liver of PGR-control piglets were significantly up-regulated (*p* < 0.05) (Figure 1N). Compared to the healthy-control piglets, BHB supplementation increased the relative expressions of *Bcl-2* mRNA (*p* < 0.05), while compared to the PGR-control piglets, BHB supplementation decreased the *Bax*, *Caspase-3*, *Caspase-8*, and *Caspase-9* mRNA expression levels, but increased *Bcl-2* mRNA levels in the liver (*p* < 0.05) (Figure 1N).

### 3.2. Changes of BHB metabolism in the liver and serum of piglets

**Figure 2:**
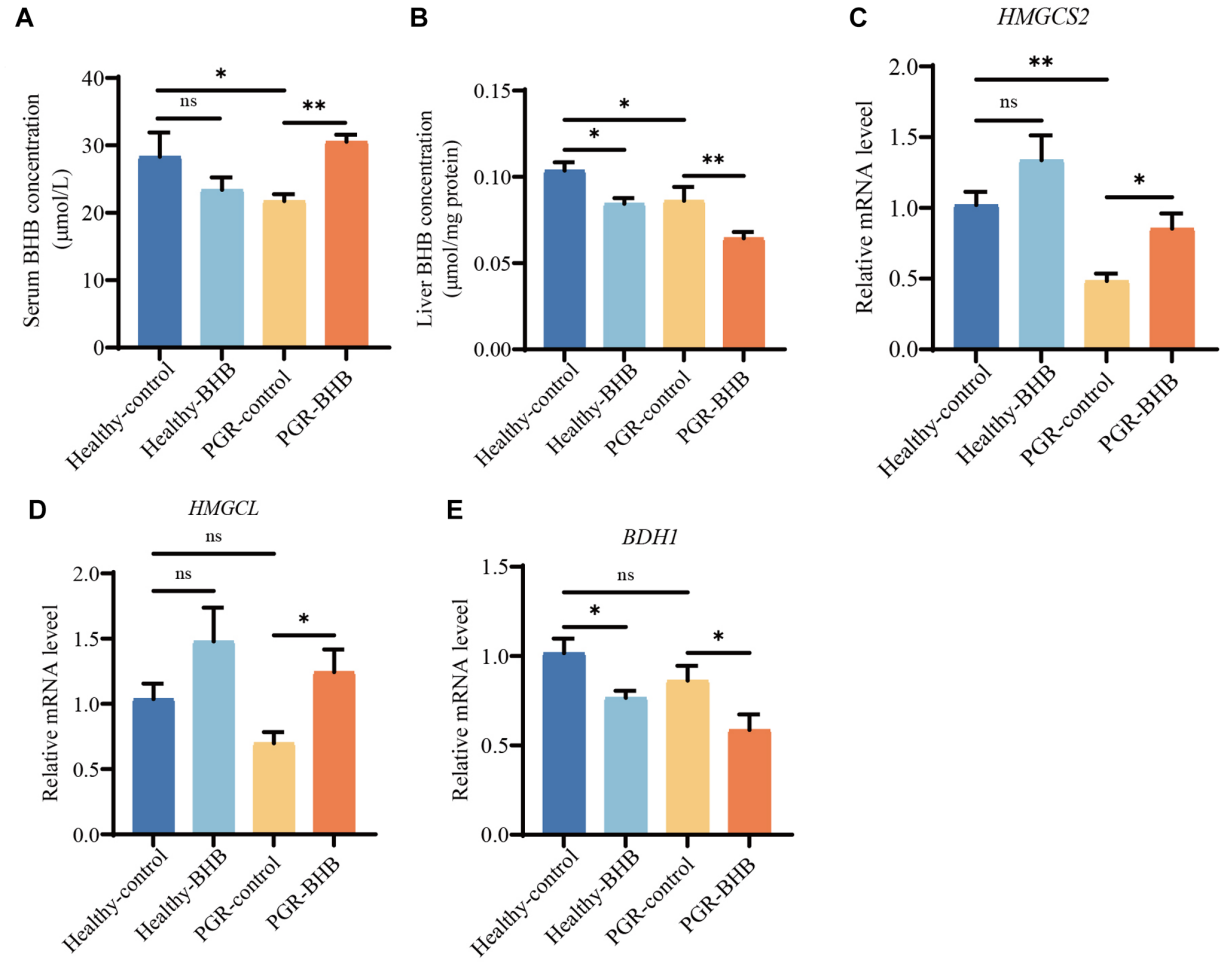
BHB metabolism of liver and serum in piglets. **(A)** Serum concentration of BHB. **(B)** Liver concentration of BHB. **(C–E)** Hepatic mRNA expression of *HMGCS2*, *HMGCL* and *BDH1*. BHB, β-hydroxybutyrate; PGR, postnatal growth retardation; HNGCS2, 3-hydroxy-3-methylglutaryl-CoA synthase 2; HMGCL, 3-hydroxy-3-methylglutaryl-CoA lyase; BDH1, 3-hydroxybutyrate dehydrogenase 1. Data were presented as mean ± SEM (*n* = 8). Different lowercase letters indicated significant differences between the compared groups (*p* < 0.05).

The BHB levels in the serum and liver of PGR-control piglets were significantly lower than those of healthy-control piglets (*p* < 0.05) (Figures 2A,B). Compared with the PGR-control group, BHB supplementation increased the serum BHB level and reduced the liver BHB content of PGR piglets (*p* < 0.05), while BHB supplementation reduced the liver BHB content of healthy piglets in comparison with the healthy-control group (*p* < 0.05) (Figures 2A,B). At the same time, the expression level of *HMGCS2* mRNA in the liver of the PGR-control group was significantly lower than that of the healthy-control group (*p* < 0.05) (Figure 2C). As compared to the PGR-control group, BHB supplementation increased the expression levels of *HMGCS2* and *HMGCL* mRNA in the liver of PGR piglets, and decreased the expression level of *BDH1* mRNA (*p* < 0.05) (Figures 2C–E). While BHB supplementation decreased the expression level of *BDH1* mRNA in the liver of healthy piglets in comparison with the healthy-control group (*p* < 0.05) (Figures 2C–E).

### 3.3. Effect of BHB on the energy tatus of PGR piglets

**Figure 3:**
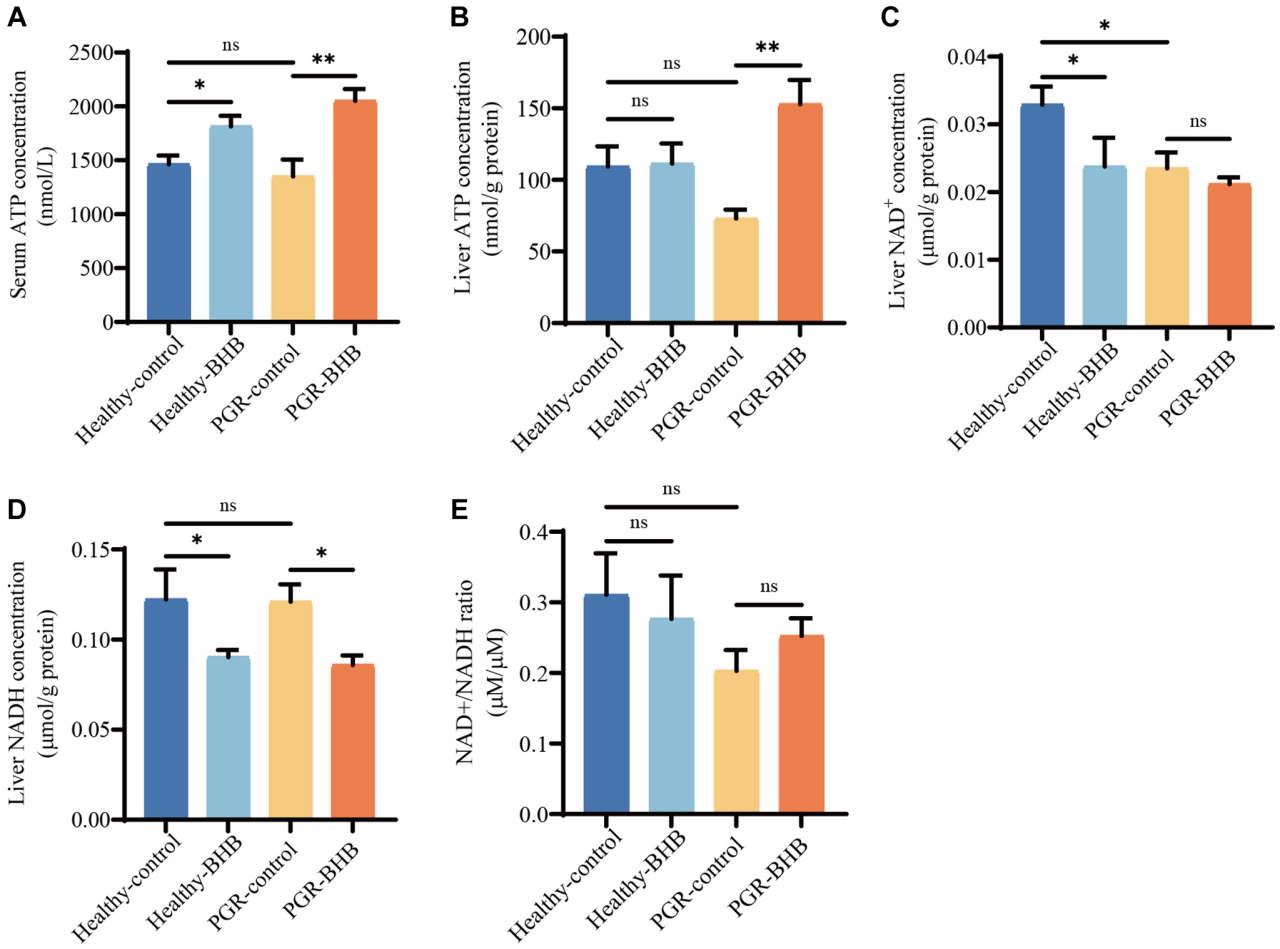
Effect of BHB on energy status of PGR piglets. **(A)** Serum concentration of ATP. **(B)** Liver concentration of ATP. **(C–E)** Liver concentration of NAD^+^ and NADH. BHB, β-hydroxybutyrate; PGR, postnatal growth retardation; ATP, adenosine triphosphate, NAD^+^, oxidized coenzyme I; NADH, reduced coenzyme I. Data were presented as mean ± SEM (*n* = 8). Different lowercase letters indicated significant differences between the compared groups (*p* < 0.05).

Compared with the healthy-control group piglets, BHB supplementation increased the serum ATP levels of healthy piglets (*p* < 0.05) (Figure 3A), while BHB supplementation increased the serum ATP levels and liver ATP contents of PGR piglets in comparison with the PGR-control group piglets (*p* < 0.05) (Figures 3A,B). In addition, the liver NAD^+^ content of piglets in PGR-control group was significantly lower than that in the healthy-control group (*p* < 0.05) (Figure 3C). Compared with the healthy-control group piglets, BHB supplementation decreased the liver NAD^+^ and NADH contents of healthy piglets (*p* < 0.05), while compared with the PGR-control group piglets, BHB supplementation decreased the liver NADH contents of PGR piglets (*p* < 0.05), but had no significant effect on NAD^+^/NADH (*p* > 0.05) (Figures 3C–E).

### 3.4. Effect of BHB on liver glucose metabolism in PGR piglets

**Figure 4:**
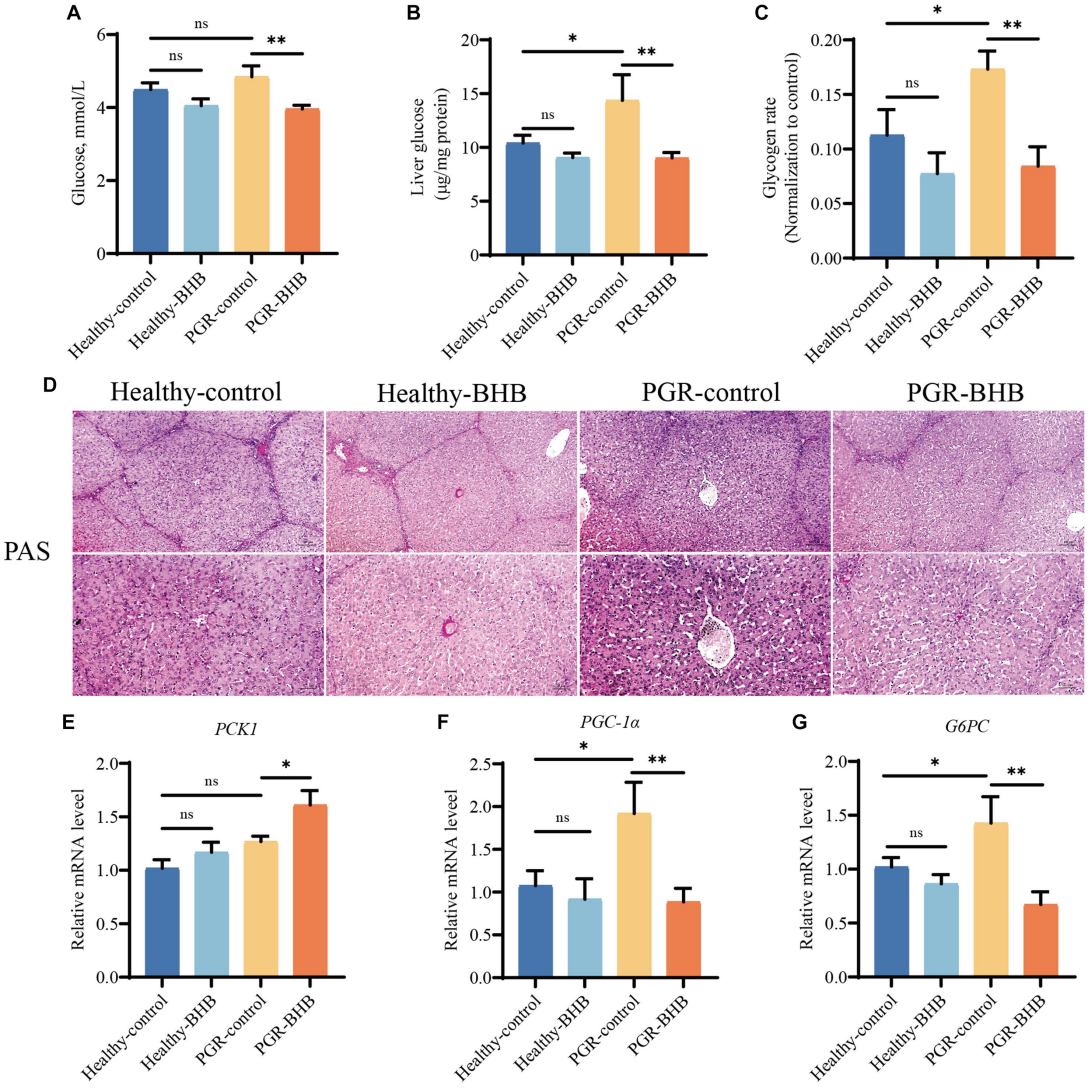
Effect of BHB on liver glucose metabolism in PGR piglets. **(A)** Blood sugar level. **(B)** Liver concentration of glucose. **(C,D)** Representative images of PAS staining and lesioned score in liver. **(E–G)** Hepatic mRNA expression of *PCK1*, *PGC-1α* and *G6PC*. BHB, β-hydroxybutyrate; PGR, postnatal growth retardation; PCK1, Phosphoenolpyruvate carboxykinase 1; PGC-1α: Peroxisome proliferator-activated receptor gamma coactivator 1-alpha; G6PC, Glucose-6-phosphatase. Data were presented as mean ± SEM (*n* = 8). Different lowercase letters indicated significant differences between the compared groups (*p* < 0.05).

Compared with healthy-control group piglets, liver glycogen content of piglets in PGR-control group were increased (*p* < 0.05) (Figures 4A–D). BHB supplementation significantly reduced blood glucose levels and liver glycogen content in healthy piglets and PGR piglets (*p* < 0.05) (Figures 4A–D) (*p* < 0.05) (Figures 4A–D). Compared with the healthy-control group piglets, the mRNA expression levels of gluconeogenesis-related genes *PGC-1α* and *G6PC* in liver of PGR piglets were increased (*p* < 0.05) (Figures 4E–G). While BHB supplementation significantly up-regulated the mRNA expression levels of *PCK1* and down-regulated the mRNA expression levels of *PGC-1α* and *G6PC* in liver of PGR piglets (*p* < 0.05) (Figures 4E–G).

### 3.5. Effect of BHB on liver lipid metabolism in PGR piglets

**Figure 5:**
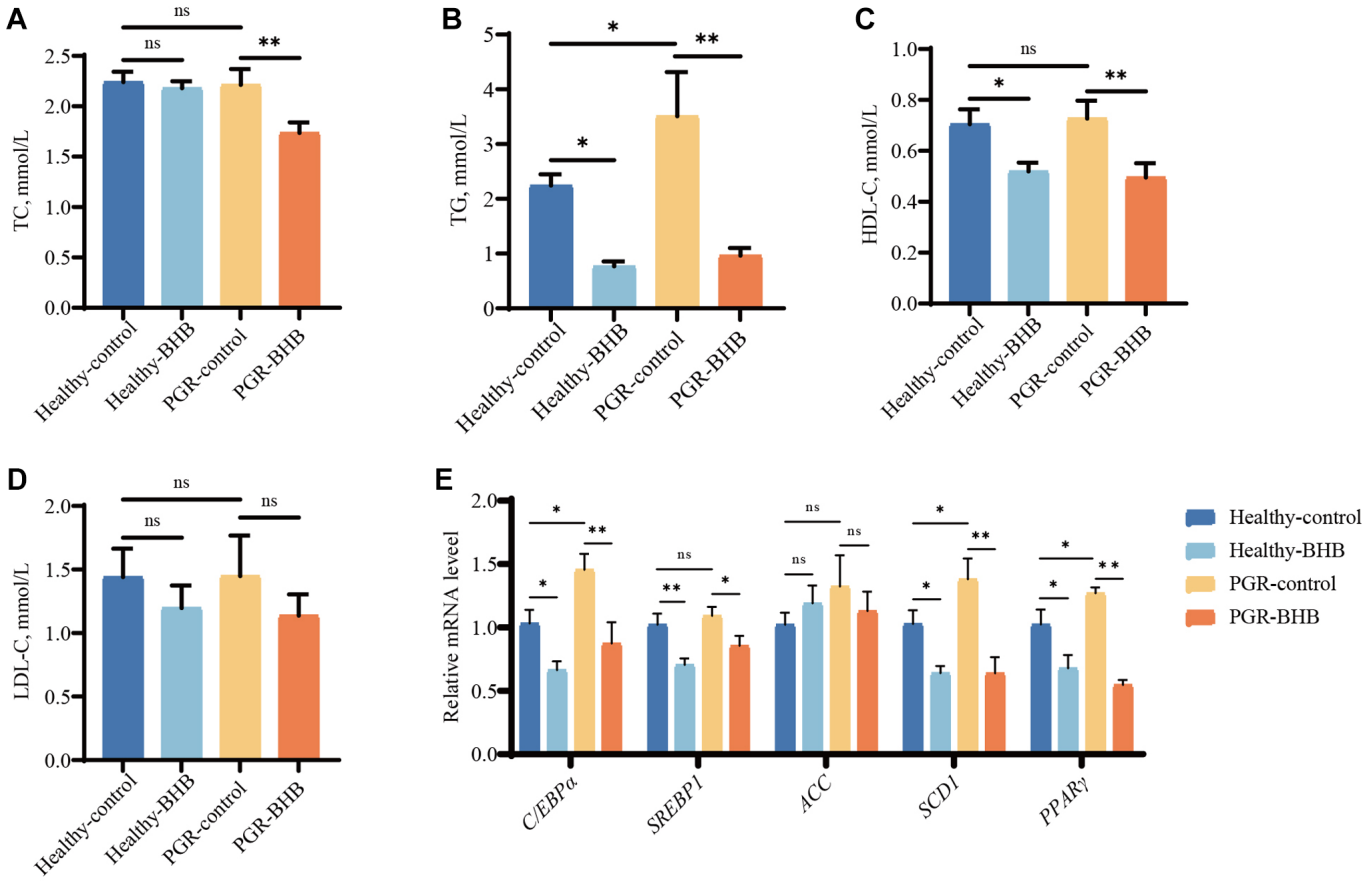
Effect of BHB on liver lipid metabolism in PGR piglets. **(A–D)** Serum biochemical parameters; **(E)** hepatic mRNA expression of *C/EBP-α*, *SREBP1*, *ACC*, *SCD1* and *PPARγ*. BHB, β-hydroxybutyrate; PGR, postnatal growth retardation; C/EBP-α, CCAAT/enhancer binding protein α; SREBP1, sterol regulatory element-binding protein 1; ACC, acetyl-CoA carboxylase; SCD1, Stearoyl-CoA desaturase 1; PPARγ, Peroxisome proliferator-activated receptor γ. Data were presented as mean ± SEM (n = 8). Different lowercase letters indicated significant differences between the compared groups (*p* < 0.05).

The serum TG levels of piglets in the PGR-control group were significantly higher than those in the healthy-control group (*p* < 0.05) (Figures 5A–D). Compared with the PGR-control group, BHB supplementation decreased the serum TC, TG, and HDL-C concentrations of PGR piglets (*p* < 0.05) (Figures 5A–D). While BHB supplementation decreased the serum TG and HDL-C concentrations of healthy piglets in comparison with the healthy-control group (*p* < 0.05) (Figures 5A–D). Compared with healthy-control group, mRNA expression levels of *C/EPP-α*, *SCD1*, and *PPARγ* in liver of piglets in PGR-control group were increased (*p* < 0.05) (Figure 5E). Meanwhile, BHB supplementation decreased the expression levels of *C/EBP-α*, *SREBP1*, *SCD1*, and *PPARγ* mRNA in the liver of healthy and PGR piglets (*p* < 0.05) (Figure 5E).

## 4. Discussion

GR is the root cause of high morbidity and mortality in infants and young children. Children with GR are more likely to suffer from metabolic diseases and pathogen infections (1). PGR occurs in 10 to 30% of piglets in the pig industry, which leads to a decrease in feed utilization and an increase in mortality, which seriously affects the efficiency of the pig industry.

Some studies have found that, compared with healthy counterparts, PGR shows poor growth performance and lower organ weight (8, 19). Consistent with the results of this study, it was found that the body weight and liver weight of PGR piglets were lower than those of healthy piglets of the same age. BHB is a water-soluble organic compound derived from lipids, which is most significantly magnified under the physiological conditions of low utilization of carbohydrates (i.e., hunger), prolonged fasting or ketogenic diet (20). Dietary supplementation of low-dose BHB can effectively improve the growth performance and organ development of early-weaned goats (21). Indeed, BHB increased the body weight of PGR piglets and improved hepatic injury and inflammatory responses in the current study. ALT and AST are considered as sensitive indicators of liver injury and are usually expressed only in hepatocytes. When the liver is damaged, they are released into the bloodstream. In this study, the activities of AST and ALT in plasma and the content of ALT in the liver of PGR piglets increased, indicating liver damage in PGR piglets, which was consistent with the results of other scholars (5, 8, 11). NLRP3 inflammatory bodies are a wide range of sensors for risk-related molecular patterns, which can be activated by many types of pathogens or danger signals (22). Pro-inflammatory cytokines (including IL-1β, IL-6, and IL-8) are necessary to initiate an inflammatory response during infection (8). The PGR piglets showed higher IFN- γ and TNF- α levels, as well as IL-8 mRNA levels in this study, suggesting that those piglets might have higher hepatic inflammation than the healthy piglets. BHB treatment could alleviate the inflammatory responses, evidenced by lower pro-inflammatory cytokines concentration and mRNA expressions, and increased anti-inflammatory cytokines levels in the liver. Consistently, *Yun-Hee Youm* et al. demonstrate that BHB can promote anti-inflammatory effects, regulate inflammation by inhibiting the activation of NLRP3 inflammatory bodies, reduce the downstream production of proinflammatory cytokines IL-1β and IL-8, and prevent NLRP3-mediated inflammatory diseases (14, 17, 18). Meanwhile, excessive inflammatory activation might be related to dysregulated apoptosis (23). It is well known that Caspase-3 and Bcl-2 are the executors and inhibitors of apoptosis, respectively. Bcl-2 is an anti-apoptotic protein that can protect cells from apoptosis, while pro-apoptotic proteins such as Bax promote programmed cell death (24, 25). Studies have shown BHB could reduce apoptosis induced by hepatic ischemia–reperfusion and paraquat challenge (14, 26). We also found that the expression of anti-apoptosis gene Bcl-2 increased and the expression of pro-apoptosis genes Bax, Caspase-3, Caspase-8, and Caspase-9 decreased in the liver of PGR piglets fed with exogenous BHB.

The ketogenic diet (KD) and exogenous ketone supplements (EKS) can increase blood BHB concentration (27–30). Increased BHB levels of serum were observed in the PGR piglets fed with BHB, while their hepatic BHB levels were decreased. This result might be explained by the feedback regulation system of ketone bodies. Ketone body synthesis in the liver mitochondrial matrix begins with fatty acid β-oxidized acetyl-CoA. Mitochondrial hydroxymethyl glutaryl-CoA synthetase (HMGCS2) condenses acetyl-CoA with acetoacetyl-CoA to form HMG-CoA, which is then released by HMG-CoA lyase (HMGCL). Most acetoacetate is further metabolized by β-hydroxybutyrate dehydrogenase (BDH1) to BHB and the activity of BDH1 is proportional to the ratio of ACAC/BHB in circulation (12, 31). The condensation of acetyl-CoA and acetoacetyl-CoA to HMG-CoA mainly depends on the mitochondrial HMGCS2, which is a rate-limiting step in ketone body synthesis (31). In the current study, the HMGCS2 and HMGCL mRNA levels were enhanced by BHB treatment in PGR piglets, while the BDH1 mRNA expression was inhibited. It is implied that BHB supplementation may alter the ACAC/BHB ratio in circulation, thereby inhibiting BDH1 activity and leading to a decrease in liver BHB content.

Liver ketone body level is strongly linked to glucose and lipid metabolism, which plays an important role in maintaining energy balance (32). ATP is the main energy source for maintaining cellular physiological responses (33). There is increasing evidence that ATP deficiency is associated with the development of liver glucose lipid metabolism disorders (34, 35). Clearly, restoring ATP synthesis in the liver may be a potential therapeutic strategy for the treatment of metabolic disorders. Some studies have found that ATP concentration in the liver of IUGR piglets and jejunum of LBW piglets is significantly decreased (36, 37), but we did not find a decrease in the liver of PGR piglets. This may be related to the acquired formation of PGR piglets. In addition, we found that administration of BHB increased the amount of ATP in the liver and blood of PGR piglets, which is consistent with the Motohisa Suzuki et al. (38) report that BHB can maintain high ATP levels. Glucose is the major energy substrate for fetal oxidative metabolism. In this study, liver glycogen production was increased and gluconeogenic gene expression was upregulated in PGR piglets, which was consistent with previous reports in IUGR (39–41). These abnormalities in glucose metabolism may be caused by insulin resistance (41). At the same time, insufficient production of ketogenic in the liver can lead to mild hyperglycemia and increased liver gluconeogenesis in adult mice (13), so increased liver glycogen production and upregulation of gluconeogenic gene in PGR piglets may also be caused by insufficient production of ketogenic. Supplementation of ketogenic precursors pantothenic acid and cysteine normalized liver gluconeogenesis in mice lacking ketogenic production (13). Our study also found that BHB supplementation can not only increase the ketone body level of PGR piglets, but also inhibit liver gluconeogenesis and reduce liver glycogen content and blood glucose concentration. Consistent with our study, Csilla Ari et al. also found that BHB could inhibit the signal of gluconeogenesis in the liver and reduce the utilization of hepatic glycogen so as to lower blood glucose levels (28, 30, 42–45). TC and TG are two key markers to reflect circulating blood lipids, which are the key steps in regulating fetal synthesis and lipid catabolism. Studies have shown that abnormal nutritional metabolism occurs in the liver of IUGR fetuses, such as abnormal fatty acid synthesis and lipid oxidation (46, 47). In this study, the serum TG level of PGR piglets was increased, and the expressions of key genes of liver lipogenesis C/EBP-α, SCD1 and PPARγ were up-regulated, which was consistent with the findings of Chen et al. (41) in IUGR mice. This abnormal elevated lipids and hepatic lipid accumulation may be due to hepatic insulin resistance (41). However, the intake of exogenous BHB significantly reduced the circulating lipids of PGR piglets, including TC, TG and HDL-C, which was consistent with the results of previous studies (28, 48, 49). At the same time, BHB can inhibit the expression of liver fatty acid synthetases SREBP1, ACC and SCD1, thereby regulating liver lipid accumulation (50, 51), which is consistent with the results of this study.

In summary, our results showed that PGR piglets had a liver injury and inflammation. Exogenous supplementation of BHB could increase the blood BHB level and regulate the inflammatory response, as well as the energy metabolism in the liver of PGR piglets. These findings provide a potential new intervention to improve liver damage and dysfunction in PGR piglets, while the underline mechanism should be further investigated.

## Data availability statement

The original contributions presented in the study are included in the article/supplementary material, further inquiries can be directed to the corresponding authors.

## Ethics statement

The animal studies were approved by all animals used in this study are humanely managed in accordance with Chinese animal welfare guidelines. The experimental scheme was approved by the Animal Protection and Utilization Committee of Hunan Agricultural University (Changsha, China; 2021042). The studies were conducted in accordance with the local legislation and institutional requirements. Written informed consent was obtained from the owners for the participation of their animals in this study.

## Author contributions

CW: Conceptualization, Data curation, Formal analysis, Writing – original draft. NW: Conceptualization, Formal analysis, Writing – original draft. YD: Methodology, Visualization, Writing – original draft. AZ: Methodology, Visualization, Writing – original draft. JL: Methodology, Visualization, Writing – original draft. BT: Conceptualization, Funding acquisition, Project administration, Writing – review & editing. MQ: Conceptualization, Funding acquisition, Project administration, Writing – review & editing. JW: Conceptualization, Writing – review & editing, Funding acquisition, Funding acquisition. YY: Conceptualization, Funding acquisition, Writing – review & editing.
